# Between mice and sheep: Biotechnology, agricultural science and animal models in late-twentieth century Edinburgh

**DOI:** 10.1016/j.shpsc.2019.01.002

**Published:** 2019-06

**Authors:** Miguel García-Sancho, Dmitriy Myelnikov

**Affiliations:** a Science, Technology and Innovation Studies, University of Edinburgh, UK; b Centre for the History of Science, Technology and Medicine, University of Manchester, UK

**Keywords:** Genetic, Embryology, Agricultural biotechnology, Animal model, Dolly, Neoliberalism

## Abstract

In this paper, we investigate the ways in which a group of scientists in Edinburgh worked across mice and sheep during the last quarter of the twentieth century. With this local episode, we show the utility of an interspecies perspective to investigate recent historical transformations in the life sciences. We argue that the emergence of animal biotechnology was the result of interactions between neoliberal policymakers, science administrators, molecular biologists, agricultural breeders, and the laboratory and farm organisms with which they worked. During the early 1980s, all these actors believed that the exportation of genetic engineering techniques from mice to farm animals would lead to more effective breeding programmes in the agricultural sciences. However, the circulation of people, money, expertise and infrastructures that the experiments required, as well as the practical constraints of working with mice and sheep, resisted a simple scaling-up from one organism to the other. This displaced the goals of the Edinburgh scientists from the production of transgenic sheep to stem cell research and human regenerative medicine. We account for this unexpected shift by looking at the interplay between science policy and its implementation via collective action and bench work across different organisms. The emergence of animal biotechnology in Edinburgh also provides historiographical insights on the birth of Dolly the sheep and, more generally, on the interactions between the molecular and the reproductive sciences at the fall of the twentieth century.

## Highlights

• The work of scientists across mice, sheep and other species provides a new lens to look at the history of biotechnology.
• Interspecies work enables historians to think differently about the interactions between developmental and molecular biology.
• Agricultural biotechnology stemmed from the collective action of different people, animals, institutions and places.
• Animals were as important as human actors in the configuration of agricultural biotechnology.
• The birth of Dolly was a contingent product of research to produce genetically modified – rather than identical – sheep.

## 1 Introduction

In June 1985, the European Commission sponsored a seminar on *New Technologies in Animal Breeding* in Edinburgh. The local organisers were the Animal Breeding Research Organisation (ABRO) and the Poultry Research Centre (PRC), two publicly-funded reference institutions with almost 40 years of experience in the field of animal genetics – eight years later, in 1993, they would both merge into the Roslin Institute, celebrated for the cloning of Dolly the sheep (Franklin, 2007; García-Sancho, 2015). The papers that were presented at the seminar captured how ongoing socio-political transformations, together with the spread of new techniques, were placing farm animal and, more generally, agricultural research at a crossroads. While some presentations addressed more traditional approaches, such as physiological, biochemical and genetic optimisation of breeding processes, others embraced recombinant techniques that allowed the direct alteration of the animals’ DNA. These techniques had been invented in the 1970s and adapted to mammals, notably mice. Their promise for agriculture was enormous and the European Communities – precursor of the European Union – were eager to catch up on a development that had mainly taken place in the United States (US).

In one of the ABRO presentations, a team led by molecular biologist Richard Lathe described what came to be known as the pharming project. This project used recombinant DNA techniques to modify sheep, so that they would produce therapeutic proteins for human consumption in their milk. The pharming project had a commercial angle, but unlike the breeding research that had taken place in ABRO so far, it was targeted to pharmaceutical companies rather than the farming sector (hence ‘pharming’ as a portmanteau of farming and pharmaceuticals). Lathe's group inspiration was the genetic modification of mice, first reported in 1980.

By the time of the seminar, several groups in the US and Europe had introduced and expressed genes from other species – viruses, rabbits, rats, and humans – in mice. These genetically modified animals were called “transgenic” (Gordon & Ruddle, 1981, p. 1244) and achieved increasing popularity throughout the 1980s. Most dramatically, in 1982 Ralph Brinster, Richard Palmiter and colleagues in the US published results of mice modified with rat growth hormone genes that grew to almost twice the normal size. With their image on the cover of *Nature* and circulating widely in magazines and newspapers, these *supermice* were the first visible testament to the power of transgenic technology. Lathe and his colleagues in Edinburgh presented their research as an “extension to farm animals” of the results successfully achieved in mice (Lathe et al., 1986, p. 91).

Mice had been used in genetics since the dawn of the field around 1900, becoming a major laboratory organism with associated knowledge, practices, and infrastructures (Rader, 2004). In the 1950s and 1960s, mouse use expanded dramatically with cancer research, and the rodents were used in huge numbers in large-scale screens of radiation, carcinogens and tumour viruses (Löwy & Gaudillière, 1998, pp. 209–249). Since their early domestication by geneticists at Harvard, Cold Spring Harbor, and later the Jackson Laboratory, their mammalian status made them a model for human health. In Jessica Bolker's (2009) terms, these were surrogate models for human health, but also exemplary models, in that they represented mammals as a whole. This status made mice ideal for research into farm animal health and breeding. Both ABRO and the PRC had incorporated lines of work on mice since very early after their creation, in the mid-twentieth century.

Historians and philosophers of biology have paid much attention to *model organisms*, a phrase that gained currency in the life sciences during the 1980s. Yet the specific practices of modelling from one organism or species to another have proved diverse and elusive. These practices of “working across species” are attracting increasing scholarly interest. They encompass actions beyond a simplistic notion of modelling, among them comparison of results (Mason Dentinger & Woods, 2018), investigation of biological processes underlying different organisms (Ankeny & Leonelli, 2011), and adaptation of medical knowledge – often, but not always, from other animals to humans (Kirk & Worboys, 2011). A common conclusion of this scholarship is that interspecies work is not a simple scalar process, and the translation of results across organisms defies naïve analogies, straightforward planning, or linear progression (Nelson, 2013; Slater, 2005). Adele Clarke and Carrie Friese (2012) have stressed that interspecies work can achieve productive results, as routine extrapolations allow for new research agendas. Scientists overcome the challenges of “transposition” from one model to another by creating dynamic relations between experimental organisms that can change along with the social world in which the research is conducted.

In this article, we investigate an unusual role for mice as models in agricultural genetics at a time of great upheaval in Britain.^1^ Recombinant DNA stirred dramatic debates, but also attracted great hopes for biological intervention and precision of genetic manipulation. In the early 1980s, it also proved a political lifeline for animal breeding, as the fledging biotechnology industry promised avenues to commercialising research, in the general context of cuts to agricultural sciences under Margaret Thatcher and neoliberal governments elsewhere (Myelnikov, 2017). Scientists at ABRO and their funder, the Agricultural Research Council (ARC), eagerly observed transgenic mice, with the pharming project being an attempt to export the technique to sheep and, eventually, other farm animals. The pharming project adopted mice as prototypes for expressing useful proteins in sheep milk.

Sheep rather than humans were thus the targets of modelling, while human patients were placed as future consumers of the resulting therapeutic proteins. We will show how the growing focus on recombinant DNA work that privileged genes and their expression as a unifying principle for biological research enabled new kinds of experiments in the field of animal breeding. These new experiments received great institutional priority and reoriented local research programmes in Edinburgh, as infrastructures that placed mice and sheep together were being improvised. In 1990, the pharming project succeeded with the birth of Tracy, one of the first transgenic sheep and the first to express large amounts of a human therapeutic protein. Yet the road to Tracy was not a clear scaling-up from mice to sheep; rather, genes, techniques, and scientists circulated back and forth across the two organisms, often leading to unexpected outcomes.

In what follows, we will address the convolutedness of this mouse-to-sheep work and present it as a novel lens to look at the history of animal biotechnology. We will argue that a main reason for this interspecies complexity was the multiplicity of actors, places, institutions and experimental organisms involved in bringing the pharming project to fruition, both at the level of scientific policy and laboratory work. The different interests and expertise operating, as well as the need to redefine infrastructures that were already in place around mice and sheep in Edinburgh, created shifts in the work across species. Sometimes, the results on sheep came quicker than those on mouse, this disrupting – and even inverting – the interspecies narrative that Lathe and his colleagues had formulated. This back-and-forth process challenged the expectation of recombinant DNA being a unifying tool for biology: instead, the circulation of this technique from mouse to sheep resembled a “dance of agency” (Pickering, 1995, p. 21ff.) between the many agendas and constraints at stake.

## 2 Biotechnology as interspecies work

By the time of the 1985 European seminar, ABRO and the PRC were facing an enormous financial and scientific uncertainty. These institutions had been founded between 1945 and 1947, in the face of the food rationing and animal disease problems derived from World War II. Over the following decades, they had consolidated as the flagship animal breeding organisations of Britain, and developed an extensive portfolio of programmes aimed at improving the commercial yield and health of cattle, pig and chicken, among other species. This leadership position had been achieved thanks to continuous funding from the Agricultural Research Council (ARC, the body of the British Government managing plant and animal research), and fruitful interactions with scientists at the University of Edinburgh (Cooke, 1981, especially pp. 277–288).

The early interactions between the Edinburgh breeding institutions and the University had been channelled through the Institute of Animal Genetics (IAG). The IAG had grown out of the Animal Breeding Research Department at the University and by the 1930s had consolidated as a leading institution in quantitative genetics – a subfield that addressed gradable characteristics of organisms, as opposed to qualitative Mendelian genetics (Button, 2017). In 1947, as ABRO and the PRC were being founded, the IAG made two key appointments in C. H. Waddington and Douglas Falconer. The former established an influential line of research that investigated the genetics of embryonic and post-embryonic development in the fruit fly, *Drosophila melanogaster.* The latter devised a methodology that enabled geneticists to predict features such as litter size in successive generations of mice. According to Falconer, there was a gradation between the organisms on which the Edinburgh geneticists worked, so that “any breeding method that might be based on the *Drosophila* results” would be tested “cheap and quick” in mice “and if it worked it could be applied with more confidence to farm animals” (Falconer, 1993, p. 139).

This led to growing collaboration between ABRO, the PRC and the IAG. The Edinburgh farm animal geneticists increasingly adopted Falconer's methodology and used mice as prototypes to design livestock breeding programmes. Mice had a shorter life cycle, “no economic value to jeopardise” and shared “a considerable inheritance with cattle, sheep and pigs.”^2^ During the post-war and Cold War years, extrapolation of mouse results led to ambitious initiatives, such as the Hereford project that explored the long-term costs and benefits of cow interbreeding at different British farms. Mice were also used as models for scrapie, a common sheep condition, in a programme developed with the Animal Diseases Research Association, a group of Scottish farmers that funded agricultural science.^3^

The proliferation of breeding research in Edinburgh – and more generally in Britain – finished rather abruptly during the 1970s. By that time, the problem of feeding the population after the War had largely been solved, and agricultural science was regarded as increasingly old-fashioned and unnecessary. In 1971, Lord Rothschild issued a report in which he urged publicly-funded research institutions to address tangible, national necessities instead of abstract academic interests. A former head of the ARC, Rothschild considered that science administration had been conducted inefficiently and moved 60% of ABRO's budget to the Ministry of Agriculture, Fisheries and Food (MAFF), where funding needed to be justified by practical outcomes (Parker, 2016; Thirtle, Palladino, & Piesse, 1997). The view that was becoming dominant in the UK was that the production of new plant and animal varieties should be left to the private sector, with state-funded institutions rather focusing on innovative breeding methods, ideally in collaboration with industry. The European Economic Communities – which Britain joined in 1973 – were by then facing food surplus issues and one of the objectives of the Common Agricultural Policy was shifting the focus from production to productivity and innovation (Garcia-Sancho, Myelnikov, & Lowe, 2017, p. 14).

The most popular innovation in the life sciences at the time was recombinant DNA, a set of techniques that was invented by a young breed of molecular biologists, with key experiments at Stanford University and the University of California, San Francisco. These technologies allowed the transfer of genetic material from one organism to another: in 1974, their inventors successfully inserted a frog gene into the bacterium *E. coli*. Soon, genetically modified bacteria were portrayed by scientists, policy-makers and commentators as potentially unlimited resources for the controlled expression of genes that produced substances of practical interest (Bud, 2010). Biotechnology start-up companies, like the Bay Area–based Genentech, sprung up to produce human insulin and somatostatin from bacteria. From the earliest days, agricultural applications were seen as an important aspect of the new techniques, and following the first experiments at Stanford the *New York Times* headline noted “animal gene shifted to bacteria: aid seen to medicine and farm” (Yi, 2015, quote from p.2).

Recombinant DNA emerged at a time in which molecular biology was seeking to expand its knowledge frontier from microorganisms to higher animals. Prior to the 1974 results, molecular biologists had built their prestige and reputation through experimental systems formed by viruses that expressed their genes in the bacterium *E. coli.* These systems were developed by Francois Jacob, Jacques Monod and other Nobel-awarded founders of molecular biology. They proved remarkably productive as “exemplars” of the biological mechanism of gene expression (Creager, 2002, ch. 8). In the mid-1970s, Jacob and colleagues shifted to multi-cellular organisms – including mice – in an attempt to address Monod's belief that “anything found to be true of *E. coli* must also be true of elephants” (quote from Monod & Jacob, 1961, p. 393; see also Morange, 2000).

This “mass migration” to higher organisms (Yi, 2015, ch. 2) was fuelled by the expectation of recombinant DNA becoming a universal tool unifying different fields of the life sciences. Molecular biologists and other life scientists believed that recombinant DNA would foster a new way of working across species, one based on the properties of genes rather than modelling or comparing biological processes. This would entail dealing with the supposedly universal language of DNA rather than having to export knowledge from the biology of one species into another: for instance, from mice to livestock in the design of breeding programmes. During the late 1970s and 1980s, biological and medical researchers enthusiastically adopted recombinant DNA, in some cases without detailed knowledge of the workings of genes at the molecular level.

Mouse geneticists and embryologists were among the most enthusiastic adopters, and Frank Ruddle at Yale University was the first to announce his team's success in inserting a herpes virus gene into a mouse genome. Ruddle's postdoc, Jon Gordon, had adapted the technique of microinjection, which the lab had been using to modify cultured cells, to introduce a DNA solution into one of the pronuclei (sperm or egg nucleus) of a fertilised mouse egg. The Yale group published in December 1980, and in the following years more experiments flooded in – the bulk of them had been initiated before Ruddle and Gordon's results. Between 1982 and 83, Ralph Brinster and Richard Palmiter of the Universities of Pennsylvania and Washington, respectively, reported the most dramatic result yet: giant mice or *supermice* were born after microinjection of their embryos with rat growth hormone gene and later human growth hormone gene (Myelnikov, 2015, ch. 4).

The extensive coverage of supermice triggered discussions about their potential. The possibility of extending the genetic modification technique to agriculture was, from the beginning, a major theme in media reports, encouraged by Brinster and Palmiter's lively speculations about the future. Speaking to *Time* magazine, Brinster said: “If we can make bigger mice we can make bigger cows” – he had trained as a vet and is based at the University of Pennsylvania's School of Veterinary Medicine.^4^ Shortly after the supermice were announced, Brinster and Palmiter started working with scientists at the US Department of Agriculture (USDA) Agricultural Research Service in Beltsville, Maryland, and in 1985 announced genetically modified rabbits, sheep and pigs. None of the foreign genes, however, seemed to make much difference to these animals (Hammer et al., 1985).

British science administrators observed the US developments with keen interest. The early-to-mid 1980s were times of concern for state-supported research institutions, following Margaret Thatcher's victory in the 1979 general election. Thatcher had been Secretary of State for Education and Science in Edward Heath's Conservative government when the Rothschild Report was implemented (Agar, 2011). Thatcher's governments shared the general rationale that had led to the commissioning of the report: the efficiency of public administration and productivity of state investment – including publicly-funded research – needed to be maximised. In that regard, the extensive breeding programmes that had characterised the UK's agricultural science and required vast amounts of land, animals and multi-year funding, did not align with the mantra of rapid delivery of results and impact on the economy or healthcare system.

Recombinant DNA offered a lifeline and an opportunity for struggling institutions at a time when the Thatcherite policies were feared, but had not yet made a significant impact on budgets. In 1979, the same year Thatcher was elected, the ARC appointed a new Secretary, Ralph Riley, who had a longstanding interest in genetic modification of plants. Riley soon explored this possibility as a more general solution for the agricultural sciences and in April 1981, months after the first Yale paper, the ARC Animal Research Station in Cambridge held a meeting on “Genetic Engineering in Domestic Animals.”^5^ One year afterwards, in 1982, the ARC published a report reviewing the strategies of two of its main research institutions, one of them being ABRO.

The ARC's belief was that rather than applying a blanket reduction of resources across its centres, it was preferable to streamline its lines of research to a few strategic areas. In its report, the ARC concluded that ABRO's “large-scale breeding experiments” were “costly and inflexible,” and that the Edinburgh institution “should not put direct effort into developing new varieties of farm animals.” The future remit of ABRO would rather become “relevant basic research” into the “genetics” of livestock using “new advancements and techniques, such as molecular biology.” It was “for these developments that additional funds would be considered” (ARC, 1982: unnumbered pages).

The ARC's conclusions suggest that the adoption of genetic engineering by British agricultural scientists was triggered by two historical processes: 1) policy reforms that discouraged the public funding of long-term breeding programmes, and 2) the expansion of recombinant DNA technologies from bacteria and viruses to mammals. Yet neither of these processes made establishing genetic engineering as a privileged line of research for agricultural science inevitable. It was the collective action of scientists, politicians and administrators at many levels – from the central government to the ARC and ABRO – that led recombinant DNA to be seen as a promising horizon that would transform the neoliberal policies into a productive rather than self-destructive scientific programme.^6^

This confluence of political and scientific agendas illustrates Barry Barnes's notion of collective agency. In a seminal contribution to the field of Science and Technology Studies (STS), Barnes argues that social and scientific change cannot simply be attributed to the sum of individual, rational choices. The transformation of knowledge systems and shared imaginaries requires the intertwined action of a variety of actors who inhabit different social worlds and whose interests coalesce in a contingent time and space (Barnes, 2000, p. 64ff). In the case of the ARC and ABRO, what transformed many isolated interests into a collective agency was the expectation that by scaling-up recombinant DNA from bacteria to mice to farm animals, it would be possible to tailor livestock breeding and make it more efficient in terms of the required resources. This aligned the agendas of policymakers seeking to rationalise R&D funding and agricultural researchers wanting to both survive and thrive as scientists in the new austerity times. Out of this alignment, the new field of animal biotechnology materialised. As the genetic engineering of farm animals became an experimental reality, the expectations and interspecies work underlying this new field were substantially reconfigured.

## 3 The pharming project across people and space

Following the ARC announcement to downsize breeding research in Edinburgh, ABRO initiated a campaign against this decision, with journalists, farming bodies and MPs expressing their outrage. This campaigning led to some concessions, and a separate channel of funding to offset the most drastic cuts (Myelnikov, 2017, pp. 716–719). Nevertheless, ABRO still lost 50% of its budget and all its experimental farms, except for a small extension of land in Dryden, seven miles south of Edinburgh. In 1983, a young reproductive physiologist, Roger Land, was appointed as ABRO's new Director. From day one, he approached the crisis as a cathartic movement that would allow the restructuring of the institutional architecture of ABRO and an emphasis on novel techniques. One of Land's first decisions was to create a molecular biology programme within which projects using recombinant DNA and related technologies could be developed.

To do this, Land built in-house expertise in molecular biology and attracted a younger generation of scientists. Shortly after his appointment, he hired Lathe, who had been employed as a researcher in one of the first European biotechnology start-ups, Transgéne SA in Strasbourg. Having worked on the development of the recombinant rabies vaccine for wild animals, Lathe had what was then rare expertise in commercial biotechnology. At Edinburgh University, Land sought the advice of John O. Bishop, then based in the sizeable Genetics Department after many years working with Waddington at the IAG. This resulted in the recruitment of Bishop's associate, John Clark, who had worked on genetic expression in mice and completed a PhD thesis on human satellite DNA (García-Sancho, 2015: 291ff).

**Fig. 1:**
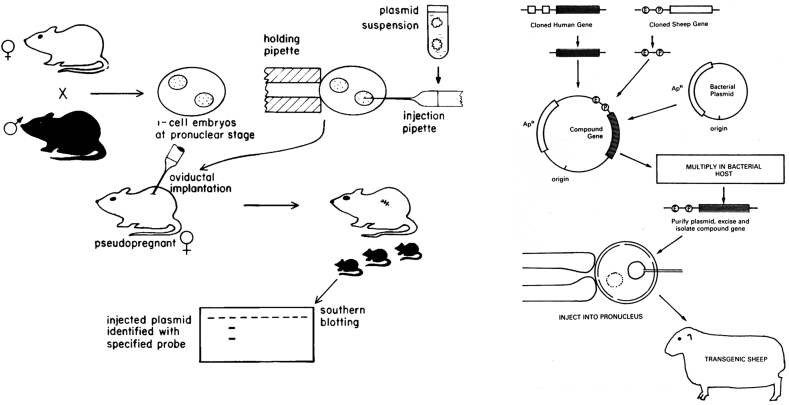
Left, Gordon's microinjection and micromanipulation system to make transgenic mice. Right, a scheme of the pharming procedure – note the similarities between both techniques. Sources: Gordon & Ruddle, 1983, p. 413, ^©^ Elsevier (left); *ABRO Annual Report—January 1985*, p.22, reprinted under the Creative Commons licence, courtesy of the University of Edinburgh Main Library (right).

Clark and Lathe initiated a line of research within ABRO's new molecular biology programme to devise an “alternative route to direct animal breeding” via the genetic engineering of “both laboratory and farm animals.”^7^ This line of research was formally launched in 1984 and became increasingly known as the pharming project. Unlike previous research in ABRO, the pharming project did not use farm animals as sources of meat, milk or wool. Building on the work across species characteristic of the biotechnology start-up companies, it sought to transform these animals into “vehicles for the production” of commercially relevant substances via recombinant DNA techniques (Fig. 1). This use of animals also distinguished Clark and Lathe's work from Brinster and Palmiter's prior production of transgenic mice and livestock: the Edinburgh researchers did not seek to augment the size of the animals, but to genetically modify sheep in order to express “human proteins of biomedical importance” in their milk (Lathe et al., 1986, p. 95). Shortly after the start of the project, Lathe left Edinburgh and Clark became the coordinator of the experimental work.

Once the experiments started, it became clear that the genetic modification of farm animals would require bridging other things apart from the mice and the sheep. The way Clark initially approached mice – as prototypes for producing transgenic sheep – was not that dissimilar to the previous use of the rodents to design breeding programmes at ABRO, despite the objectives being different. Nicole Nelson has shown that using animals in this prototypic way – to model processes that may or may not be exportable – involves moving “from the specific to the specific” rather than “from the specific to the general or the simplified to the complex” (Nelson, 2013, p. 7). Soon after the launch of the pharming project, Clark realised that the success of the experiments depended on the creation of an infrastructure – of people, laboratories and technologies – that could account for the genetic modification of *both* mice and sheep. This infrastructure initially emerged from the pre-existing space of animal breeding in Edinburgh.

**Fig. 2:**
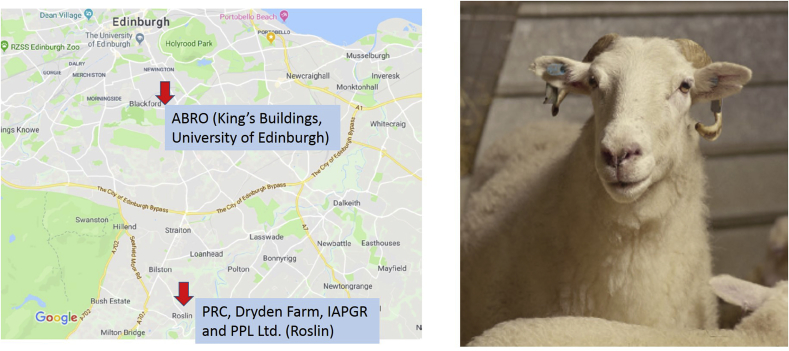
Left, map with the location of the different Edinburgh breeding institutions (elaborated by authors from Google Maps). The Cambridge Research Station of the IAPGR was located in Babraham, 350 miles south from Edinburgh. Right, Tracy, the first successful transgenic sheep born in Roslin in 1990 (courtesy of the Roslin Institute, reprinted with permission).

For the first five years of the project (1984–89), the mouse side of the work relied on the University's animal house. This facility had been used in the previous breeding research and was located within close distance of ABRO's headquarters, based in the University's life sciences campus – the King's Buildings – in the southern suburbs of Edinburgh (Fig. 2). With the start of the pharming project, the animal house became a space of convergence of different mouse experiments, with Clark's standing as a member of a new generation of geneticists effectively mediating between ABRO's past allies at the University – focusing on Falconer's predictive methods – and those wanting to venture into genetic modification.

Genetically modifying sheep, as opposed to mice, required even rarer expertise in developmental biology. These skills came from within ABRO, in the figure of Ian Wilmut, a reproductive physiologist with considerable experience in embryo transfer. Wilmut had joined ABRO in 1973, after training and working at the ARC Animal Research Station in Cambridge. His appointment to the new project was not smooth: to his frustration, Wilmut was forced away by Land from his earlier work on embryonic mortality in sheep, and asked to help Clark adapt the genetic engineering system from mouse to farm animals.^8^

Clark's mouse experiments and Wilmut's research on sheep effectively divided the pharming work between the University's animal facilities and Dryden farm, located five miles apart (Fig. 2). The institutional space of Edinburgh was further reconfigured when, in 1986, the Agricultural and Food Research Council (AFRC, as ARC was renamed in 1983) forced ABRO to merge with two other institutions in a move that sought to streamline scientific programmes and administrative costs – other AFRC centres were closed altogether. These institutions were the PRC and the Institute of Animal Physiology in Babraham (Cambridgeshire).^9^ The resulting organisation was named the Institute of Animal Physiology and Genetics Research (IAPGR), a large conglomerate with two geographically distant stations, one around Cambridge and the other around Edinburgh. The Edinburgh Research Station was housed in the old PRC headquarters at Roslin. However, Clark and his collaborators did not move until the Roslin facilities opened its own animal house and mice lived in proximity to rats, rabbits and sheep on the Dryden farm.^10^

In 1987, IAPGR researchers, aided by the Scottish Development Agency, secured investment for Caledonian Transgenics Ltd. – soon renamed Pharmaceutical Proteins Limited (PPL) – a start-up biotechnology company to market the deliverables of the pharming project. The company provided funding to the project in exchange of exclusive rights over the therapeutic proteins that the transgenic sheep would produce. These proteins were then expected to be sold to pharmaceutical companies which would transform them into drugs (Clay & Goldberg, 1997). PPL worked in close association with the pharming team, but independently from the IAPGR, hiring its own researchers and leasing the Edinburgh Research Station's infrastructure.^11^ The rest of the funding to pharming came from the Transgenic Animal Programme, a grant scheme that the AFRC established one year afterwards with IAPGR researchers becoming especially successful applicants.

All these scientific and institutional moves show that by the late 1980s, animal biotechnology had shifted from a term in a policy report to a set of projects, one of which was developed by a team of people working in different places at Edinburgh. The pharming project had expanded the collective agency within which animal biotechnology originated in Britain, and transformed it into an experimental programme that went far beyond a simple bridging – or scaling-up – of genetic engineering from microorganisms to mice and sheep. STS literature has shown how interspecies work can significantly remake geography. Friese and Clarke (2012) stressed the importance of “transposing” infrastructures across species, via the movement of techniques, or the alliance – or friction – between different people. In these processes of transposition, species do travel across space, understood as both a physical territory and in the more metaphoric meaning of a disciplinary field or funding scheme (see also Davies, 2012; Milne, 2012).

In Edinburgh, the work of exporting recombinant DNA from mouse to sheep required the collective action of scientists coming not only from molecular biology, but also from experimental embryology. This collective action entailed both collaboration and dissent, as exemplified by Wilmut's enforced move to the pharming project. Moreover, the production of genetically modified mice and sheep triggered the transposition of infrastructures that both ABRO and the University of Edinburgh had created around both organisms over the preceding decades. The new institutional setting within which the experiments took place was spread across places that were distant in both a geographical and more figurative sense: a biotechnology start-up company, a university animal facility and a research institute with dedicated government funding for transgenic work and two stations more than 350 miles apart. The mice and sheep with which the Edinburgh researchers worked added further complexity to this assemblage.

## 4 Back-and-forth between organisms and disciplines

The first protein that the pharming team targeted for commercial development was alpha-1-antitrypsin, which is used in the treatment of emphysema and cystic fibrosis. Other laboratories in Britain – notably University College London – were working with the gene that encodes the protein and the team relied on the existing networks of gene exchange, as well as their own efforts, to obtain and optimise the necessary DNA. Clark's plan was to test the insertion of the human gene that expresses the protein in mice, select the best gene constructs and inject these into sheep embryos.^12^ However, this initial strategy broke down, as alpha-1-antitrypsin did not behave predictably in mice, and the parallel sheep work developed faster – especially in the expression of sufficient levels of protein for therapeutic use. This led the pharming project to its first striking results with the birth of Tracy.

Tracy produced immense amounts of alpha-1-antitrypsin in its milk and was born in the Dryden farm in 1990, the same year that the full pharming team moved to the IAPGR facilities in Roslin (Wright et al., 1991). While not the first transgenic sheep made in Edinburgh, she was the first clear success and was widely promoted as such (Fig. 2). For the first time, the importance of an Edinburgh-bred animal was founded on the human and medically relevant gene it carried rather than on Tracy being a sheep or having special qualities for the production of food or wool.^13^ It was thus a clear victory for the pharming project, and one that highlighted the new goals that the Edinburgh scientists were pursuing through interspecies, mouse-to-sheep work.

Despite the success of Tracy, this mouse-to-sheep work was becoming increasingly convoluted. Nelson has investigated the use of mice as models for human behaviour and proposed the notion of “epistemic scaffold” to conceptualise the inferences that researchers make across species. Since both mouse and human are highly specific organisms, they require knowledge-generation mechanisms that are flexible and can be modified over time. Epistemic scaffolds are protocols aimed at generating definite and enduring knowledge; yet, they can be dismantled and reconfigured at will when new findings alter the relationship between one organism and the other (Nelson, 2013: 7ff). Nelson's concept aligns with Friese and Clarke's notion of transposition, which captures the continuous, back-and-forth movement of knowledge from one organism to another. In this circulation, the similarities or generalisations on which the modelling relationship is founded often need serious qualification or dismissal. However, establishing a connection between the two organisms is still a highly productive strategy and may persuade funders of the potential of an emerging field (Friese & Clarke, 2012, p. 36ff).

These highly volatile, but ultimately productive processes of knowledge circulation were all at play in the pharming project, where work leading to transgenic mice provided the inspiration for the genetic modification of sheep. More generally, the hypothesis of scaling-up genetic engineering between these two organisms was a crucial trigger for the collective agency that resulted in the emergence of animal biotechnology and the AFRC Transgenic Animal Programme. Yet at the bench level, the epistemic relationship between the two organisms was continuously reconfigured and, at some points, inverted in Edinburgh: when sheep expressed alpha-1-antitrypsin better than mice. Since the sheep became a reliable organism for molecular intervention in its own right, mouse and sheep work turned increasingly independent at the IAPGR, and only connected to address specific experimental problems.

One such problem was the inefficiency of microinjection in both mouse and sheep. The success rate of injecting foreign genes into the mouse pronuclei had been low since Gordon's initial experiments at Yale, and in a well-established lab, the proportion of isolated embryos that resulted in live transgenic mice was 2% (Camper, 1987). The team that made Tracy reported an even smaller 0.91% success rate with sheep (Wright et al., 1991, p. 831). Taking a much longer pregnancy and higher cost into account, a more efficient way of making transgenic sheep was a priority. In this case, mouse work paved the way: while the prototypic relationship between mice and sheep may have been challenged by the success of Tracy, the more expansive and well-funded mouse research still served as a major inspiration for what was possible, scientifically exciting, and a desirable goal.

Since the early 1980s, an alternative way of producing transgenic mice had been the use of stem cells that could be genetically modified in culture and introduced into the embryos. This line of research had originated in cell biology laboratories, with *embryonic* mouse stem cells being isolated in Cambridge by Martin Evans and Matthew Kaufman, and independently by Gail Martin at University of California in San Francisco (Evans & Kaufman, 1981; Martin, 1981; see also Lancaster, 2017). Their pluripotency was a key feature: due to these being undifferentiated cells, they could contribute to the embryo's germline and thus whole mice could be bred from them. Crucially, this meant that genetic modification could be done in the cells in vitro, with the kind of selection systems used in bacteria and somatic animal cells – one no longer had to hope that the gene would interact as expected in an embryo. Towards the late 1980s, genetic modification of stem cells was thus becoming a promising tool to improve control over inserted genes, as well as the success rate of transgenesis.

Some IAPGR researchers, especially those with a cell biology background, attempted to genetically modify animals via stem cells. Jim McWhir, a former PhD student of Evans, had moved to Edinburgh as a postdoctoral fellow and obtained his first grant from the Medical Research Council to genetically engineer embryonic stem cells in mouse and explore their potential therapeutic properties (Myelnikov & Garcia-Sancho, 2017, p. 8). In the mid-1990s, he associated with Wilmut to assess the potential of stem cells in the genetic modification of sheep embryos – the AFRC Transgenic Animal Programme accepted applications for stem cell work. However, unlike with alpha-1-antitrypsin, embryonic stem cells were more difficult to isolate in sheep than in mice.

This led Wilmut and McWhir to explore nuclear transfer as an alternative approach. Like stem cells, this technique had previously been used in embryology and cell biology laboratories with mice, even if cloning mice through nuclear transfer – reported by Karl Illmensee in 1981 – was later dismissed as fraudulent (Kolata, 1997, pp. 103–133). Nuclear transfer involved the creation of an artificial embryo by extracting the nucleus of a cell and inserting it into an enucleated oocyte – an unfertilised egg that had been previously devoid of its own nucleus. If the new, inserted nucleus belonged to an adult cell and the resulting embryo was allowed full-term development, the offspring would be genetically identical to – a clone of – the cell donor.

The first candidate cell nuclei for nuclear transfer in Edinburgh were those that McWhir was exploring in his search for embryonic stem cells. They were sheep embryo nuclei and showed some plasticity, despite being more differentiated and less pluripotent than the elusive stem cells. The team decided that the first nuclear transfer experiments would be conducted in the absence of any genetic modification and, in 1995, Megan and Morag were born in Roslin after the insertion of McWhir's nuclei into an enucleated oocyte (Campbell, McWhir, Ritchie, & Wilmut, 1996). One year afterwards Keith Campbell, a recent team recruit and an expert in developmental cycles, suggested to repeat the experiment inserting an adult – rather than an embryo – cell nucleus into the oocyte. This led to the birth of Dolly, the first mammal that was genetically identical to another adult sheep – the donor of the cell nucleus (Wilmut, Schnieke, McWhir, Kind, & Campbell, 1997). Both of these achievements occurred in a renewed institutional setting, since in 1993 the Edinburgh Station of the IAPGR had split with Babraham and become again an independent institution called the Roslin Institute.

Dolly, Megan and Morag multiplied the layers of complexity of the mouse-to-sheep work in Edinburgh. Stem cells and nuclear transfer derived from a different tradition than transgenic technologies, one that was less connected with altering the structure of genes, and more with the replication of the DNA molecule in the process of cell and embryo division. Wilmut, McWhir and Campbell came from an embryology and cell biology background rather than molecular biology. In producing the three cloned sheep, they originally modelled their work on mouse, as they had done in the previous case of Tracy. However, like in Tracy's genetic modification, they moved beyond the state-of-the-art of research on this organism: the dominant view among life scientists in the early-to-mid 1990s – especially after Illmensee's fraud allegations – was that successful nuclear transfer would be difficult, if not unachievable in mammals (Myelnikov & Garcia-Sancho, 2017, pp. vi-vii and 1). With the cloning of Megan, Morag and Dolly, sheep work in Edinburgh moved faster than mouse work.

The planning of the pharming project was to bring together these different layers of mouse-to-sheep work by recasting stem cells and nuclear transfer as tools for genetic modification. Following the birth of Dolly, Wilmut, Campbell and McWhir would produce a transgenic sheep by transferring to the enucleated oocyte a genetically modified rather than intact nucleus, ideally from an embryonic stem cell. This would improve the efficiency of sheep production and, once the successful genetically modified sheep were born, the cloning technology could be used to perpetuate this animal, through the same procedure that had led to Dolly. At a broader disciplinary level, producing a cloned and genetically modified sheep, or cloning a successful transgenic sheep, would subordinate the embryological and cell biological techniques to the objectives of molecular biology, thus confirming recombinant DNA as a universal tool unifying different fields of the life sciences.

Six transgenic and cloned sheep were born in Edinburgh in 1997, one year after the birth of Dolly. The most promising of them was named Polly and incorporated into her DNA the gene coding for the human protein factor IX that helps in the treatment of haemophilia (Schnieke et al., 1997). There were yet some factors that led Polly to receive far less public attention than Dolly, even in the internal publicity of the Roslin Institute. Firstly, the genetic modification could not be conducted in embryonic stem cells, that had remained elusive for sheep. In that instance, Wilmut, Campbell and McWhir's work always ran behind that of mouse researchers. Secondly, and more importantly, the levels of expression of factor IX were insufficient for an industrial development: PPL did try to market this protein and Tracy's apha-1-antitrysin, but neither product completed clinical trials.^14^

Scholars have documented a long and established tradition of work across species in the reproductive sciences, one that is at least as prolific as molecular biology (Davies, 2012; Hinterberger, 2018). Friese and Clarke have shown that work across species around animal reproduction is populated with “sources of recalcitrance” that prevent the results from one organism to be generalised to the other. Far from obstructing transposition, Friese and Clarke argue that those recalcitrant processes can be enormously creative, leading research to “new opportunities” and different “sets of questions” (Friese & Clarke, 2012, p. 34 and 46). The Edinburgh scientists successfully transposed the genetic modification and nuclear transfer technologies from mouse to sheep – they actually overcame some of the sources of recalcitrance derived from the prior adaptation of these technologies from microorganisms to mice. However, the commercial expectations attached to this transposition were never met: the cloned and transgenic sheep did not enable PPL to market therapeutic proteins. In other words, the exportation of recombinant DNA and nuclear transfer from mouse to sheep did not effectively scale-up commercial biotechnology from microorganisms to farm animals.

This led Wilmut, Campbell and McWhir to look for alternative objectives and independent funding. The pharming project's core work on genetic modification had always been supported by the AFRC – known as the Biotechnology and Biological Sciences Research Council (BBSRC) since 1994. However, ahead of the nuclear transfer experiments, Wilmut applied to and obtained a grant from the Ministry of Agriculture, Fisheries and Food (MAFF). MAFF had been an important contractor of the Edinburgh institutions since the time of Rothschild's reforms. Its political agenda differed substantially from that of the AFRC and BBSRC in that MAFF was eager to distance itself from any genetic engineering work, which was too market-oriented and, crucially, controversial at a time in which genetic modification of food – the GM crops – was increasingly questioned.

MAFF authorities went as far as suggesting any reference to making transgenic sheep via nuclear transfer be removed from the Dolly paper draft as well as press releases, and the reproductive potential of cloning be emphasised instead. Thus, shortly before Dolly was announced to the world, Wilmut's MAFF liaison queried

“What is meant by the sentence ‘Gene targeting in livestock will become possible by nuclear transfer from transformed cell populations’[…]? MAFF does not support research to produce transgenic livestock animals and the tone of this sentence suggests that the production of livestock animals might be an aim of your work”.^15^

As a result of these pressures, as well as the general fascination with cloning, the Roslin scientists presented Dolly as an end in itself in both the scientific article and press releases. This led scientific commentaries and media reports to portray Dolly as a cloned animal rather than a vehicle for genetic modification (Brown, 2000; Holliman, 2004). The line of research that had led to the nuclear transfer experiments was consistently omitted or downplayed in most accounts of the birth of Dolly. This multiplied the gap between the reproductive scientists and the core objectives of pharming.

The growing divergence of Dolly's creators from the idea of pharming moved the project to unexpected territories. Andrew Pickering has accounted for these serendipitous trajectories in an influential essay where he compares scientific practice with a “dance; ” one partner is represented by the researchers equipped with technologies and the other by the “material agency” of the natural world. The dance triggers continuous, reciprocal and, crucially, unpredictable interactions, since the behaviour of organisms and other entities from the natural world cannot be fully predicted or channelled. Scientific practice is consequently shaped – or mangled – through bidirectional encounters between human and material agency: on the one hand, the objectives of scientists and the (limited) power of their technologies, and on the other hand the constraints imposed by the natural world. Time is a crucial variable in this process, since the “mangle of practice” occurs during the course of projects and leads the goals that were originally written in research proposals to be constantly reformulated (Pickering, 1995, p. 21).

In Edinburgh, the material agency of mice and sheep – embodied in their different biology – qualified the pharming project's objective of scaling-up commercial biotechnology to farm animals. In this qualification, a multiplicity of actors – not only scientists and technologies – intervened, among them media, their audiences and funders such as MAFF, with markedly different interests from the BBSRC. This growing conglomerate of animals, technologies, individuals, places and institutions exerted a collective agency that differed from the one that had resulted in the original formulation of animal biotechnology by the ARC and ABRO: it prioritised cloning and stem cells over the production of transgenic sheep. In Friese and Clarke's terms, a recalcitrant process led the practice of nuclear transfer to acquire a life of its own and be creatively recast beyond the objective of genetic modification.^16^

This new life of cloning gradually displaced the idea of pharming. In 1999, the Roslin Institute received a six-year grant from the US pharmaceutical company Geron to investigate the production of human and animal stem cells. Geron was also a funder of a group at the University of Wisconsin that had reported the isolation of the first stem cells from human embryos (Thomson et al., 1998). Human embryonic stem cells were highly pluripotent and could regenerate damaged tissues, especially if they were obtained via cloning: a cell nucleus of the patient was inserted into an enucleated oocyte and transformed into an embryo from which the stem cells were extracted. This placed Roslin's nuclear transfer technique as an ideal source of patient-specific stem cells and led Geron to buy the patent in exchange of the grant. Such level of funding crucially alleviated the chronic financial problems of the agricultural sciences in Edinburgh, but at the price of shrinking their original animal breeding remit.^17^ Even today, Roslin scientists and most biotechnology commentators still see the production of stem cells as the main legacy of Dolly, while the original pharming project has largely fallen into oblivion.

## 5 Conclusion

Historians have paid increasing attention to the tradition of animal breeding in Edinburgh and, more specifically, the line of research on genetic engineering within which Dolly was born (Button, 2017; García-Sancho, 2015; Myelnikov, 2017). In this article, we have argued that the genesis of that line of research – called the pharming project – and its transition from animal transgenesis to human regenerative medicine can only be fully captured by looking at how the Edinburgh scientists worked across mice and sheep during the last quarter of the twentieth century. More fundamentally, and in line with existing historiography (Kirk & Worboys, 2011; Mason Dentinger & Woods, 2018), this mouse-to-sheep perspective offers new insights on the ways biology and medicine, human and animal health, and reproductive and molecular science interacted during the 1980s and 1990s, a time of financial uncertainty for agricultural research.

The will of the pharming scientists to export genetic modification technologies from mouse to sheep both built on a longstanding tradition of work across these two organisms in Edinburgh and reconfigured existing objectives of interspecies work. In this reconfiguration, the mice and the sheep were as important as the stated objectives of the project: diverging experimental results across these two organisms challenged modelling assumptions that were based on the imagined capacity of recombinant DNA to bypass biological barriers. Laboratory and farm animals formed a conglomerate with neoliberal policies, science administrators and biological researchers from many fields. All these actors exerted a collective and historically contingent agency that shaped the identity of animal biotechnology: whereas in the 1980s this identity was more oriented to genetic modification, it gradually shifted towards stem cells and regenerative medicine. The resistance of the organisms to over-simplistic modelling and the entrance of new actors into the conglomerate – from places as diverse as the media and biobusiness – were key to this reorientation.

Investigating animal biotechnology from an interspecies perspective provides a new lens to analyse the birth of Dolly. Sarah Franklin borrowed “the very conservative idiom of genealogy” to argue that “Dolly came onto the scene for a whole flock of reasons that connect her, Roslin, bioscience, cloning, stem cells, and a myriad other biocultural entities together through lineages that are familiar, and even traditional, but newly hybridized, or mixed” (Franklin, 2007, p. 13). By inserting mice into the equation and looking at how this organism inspired experimental work on sheep, we have proposed the pharming project as a complementary narrative, one that does not place Dolly at the centre of the stage, neither makes her birth be regarded as inevitable. Yet the relationship of the pharming project with Dolly is not as clear as it may seem at a first glance. If we interpret that pharming finishes with the genetic modification of sheep, Dolly would be just one of the constitutive genealogies of this project, along with Thatcherite policies, genetic engineering techniques and the reconfiguration of the agricultural sciences. If we alternatively identify stem cell research as the most definite – and successful – outcome of the pharming idea, Dolly would be the turning point that reconfigured the fate of the project. In all scenarios, the birth of Dolly appears as a contingent phenomenon, the product of a broader line of research rather than the primary or sole objective of scientists.

At a more general historiographical level, the reconfiguration of animal breeding in Edinburgh challenges existing assumptions about how molecular and developmental biology interacted at the fall of the twentieth century. Historians have argued that the transition of experimental research from microorganisms to higher organisms was the main reason why molecular biologists incorporated embryological perspectives from the mid-1970s onwards (e.g. Morange, 2000). Our story suggests that, in this incorporation, embryologists were as proactive as molecular biologists and showed both the limitations of and alternatives to a purely genetic approach. Ian Wilmut, Jim McWhir and Keith Campbell were not involved in the original pharming idea and incorporated a different way of working across mice and sheep, one that was based on stem cells and nuclear transfer instead of genetic engineering techniques. The lasting legacy of Dolly over Polly suggests that despite the promise of genetic engineering, cell and developmental biology also exerted a lasting influence within and beyond agricultural science, in fields such as regenerative medicine. Our paper, consequently, chimes with other historians who have stressed the importance of the reproductive sciences in the making of biotechnology, a field commonly – and narrowly – associated with molecular biology (Wilmot, 2007, pp. 303–315). We also align with recent proposals of investigating the history of twentieth century agricultural science beyond a narrow focus on genetics (Meunier & Nickelsen, 2018).

In 2008 Wilmut, considered by many the father of Dolly, announced that he would stop using nuclear transfer in the face of new advancements that had made this technique unnecessary to obtain patient-specific stem cells. That same year, he became the founding director of the Centre for Regenerative Medicine, a new Edinburgh-based institution supported by the Medical Research Council rather than the Biotechnology and Biological Sciences Research Council, the traditional funder of the Roslin Institute and all its predecessor institutions. This shows that by the early twenty first century regenerative medicine had established itself as an independent field from the tradition of animal genetics and agricultural research in Edinburgh. Our paper has reconstructed this connection by exploring how Wilmut and his colleagues worked across mice, sheep and, ultimately, humans. This has enabled us to offer an account that historicises the birth of Dolly and, at the same time, raises a new perspective on the emergence of animal biotechnology.

## Footnotes

1 Our main source is the recently available archive of the Roslin Institute and its predecessor institutions at the University of Edinburgh: https://collections.ed.ac.uk/towardsdolly/catalogues (last accessed January 2019). Historians and social scientists have amply investigated the use of mice, but often restricting their analysis to scientific fields in which the rodents modelled human processes. By looking at work across mice and other non-human organisms, we align with appeals to overcome the anthropocentrism of current literature (Kirk & Worboys, 2011; Mason Dentinger & Woods, 2018).

2 N. Bateman, “ABRO's mice,” *ABRO Annual Report – January 1967*, pp. 33–40 (quotations from p. 33-4). Edinburgh University Main Library, Special Collections, Records of Roslin Institute and Predecessor Institutions, EUA IN23/1/1/2.

3 C.G. Dickinson, “Catching (up with) scrapie,” and St. C.S. Taylor, “A Multibreed approach to Breed Comparison,” *ABRO Annual Report – January 1972*, pp. 7–13 and 15–20. Edinburgh University Main Library, Special Collections, Records of Roslin Institute and Predecessor Institutions, EUA IN23/1/1/2. On ABRO's research on scrapie, see also Kim, 2007, ch3.

4 “Mighty Mice: Gene transfers create giants”, *Time*, 16 December 1982, p. 53. See also J. Leith, “The Livestock Industry's Genetic Revolution,” *Business Week*, 21 June 1982, 124–32.

5 ‘Conference Literature, 1981‘, Add. 8388, Anne McLaren Papers, The British Library (London).

6 For a discussion on how to navigate this multiplicity of actors and interests in the history of recent biology see García-Sancho, 2016. The annual research reports on which this article is based combine accounts written by both administrative managers and scientists in charge of the experimental work.

7 “Research Programme”, *ABRO Annual Report -- January 1984*, p. 42. Edinburgh University Main Library, Special Collections, Records of Roslin Institute and Predecessor Institutions, EUA IN23/1/1/2.

8 This adaptation required complex micromanipulation experiments: due to a hand tremor, Wilmut was assisted in the practical aspects of sheep embryo microinjection by a recent ABRO recruit, Paul Simons (Myelnikov and Garcia-Sancho, 2017, p. 2).

9 For further discussion on breeding research in the Cambridge area see Polge, 2007.

10 B. Cross to R. Land, 21th October 1986. File relating to the new mouse accommodation on the Roslin site. Edinburgh University Main Library, Special Collections, Records of Roslin Institute and Predecessor Institutions, EUA IN23/3/5/5/1. John Bishop and Richard Lathe, oral history conducted by D. Myelnikov on 1st March 2016 in Edinburgh.

11 Shortly after the launch of PPL, in 1988, Thatcher's government suddenly discouraged any public investment in what was defined as ‘near-market’ research – a form of applied work that was too close to commercial viability to receive state support (Myelnikov, 2017). PPL squared with this new imperative of moving a substantial part of the funding of applied research to the private sector and fitted with the way animal biotechnology was being configured in Britain: in the face of Rothschild and Thatcher's policies, investors and entrepreneurs regarded medicine as a safer area than agriculture.

12 To conduct the genetic modification experiments, Clark needed the contribution from Alan Archibald, a biochemist who in the hectic years preceding the AFRC's cuts had visited Heidelberg to train in recombinant DNA technologies at the European Molecular Biology Laboratory. Archibald carried mice with the alpha-1-antitripsin gene on the train from London to Edinburgh (interview with A. Arcbibald by G. Bulfield. Edinburgh University Main Library, Special Collections, Records of Roslin Institute and Predecessor Institutions, EUA CA15/5; Myelnikov and Garcia-Sancho, 2017, pp. 5–6).

13 Social science literature has shown that modern industrial farming renders animals “invisible” through standardisation processes that often involve genetic selection and modification (Lonkila, 2017).

14 The site of integration of the foreign human gene into the sheep's genome, as well as post-translational modifications, left the levels of protein expression largely unpredictable. This, together with the difficulties of producing viable transgenic sheep, created uncertainties that complicated PPL's commercial developments. Other companies outside Edinburgh successfully manufactured products via transgenic animals: an early example of this is Herman the bull, produced by the Dutch-US firm Pharming in 1990 (Myelnikov and Garcia-Sancho, 2017, 7 and 27ff.).

15 Letter from MAFF to Ian Wilmut, 7 November 1996, The National Archives of the UK (TNA): Ministry of Agriculture, Fisheries and Food: Animal Science and Biotechnology: Registered files (BUB prefix): Development potential of quiescent cells derived from sheep embryos, sheep foetuses and adult sheep: Roslin Institute research project LS1202, MAF 662/1.

16 For similar *dances of agency* in which the behaviour of non-human organisms leads scientists to redefine research objectives see Coppin, 2008 and Nickelsen, 2017.

17 “Roslin signs six year deal with Geron,” Roslin Institute Annual Report, 1998–99, pp. 14–17. Edinburgh University Main Library, Special Collections, Records of Roslin Institute and Predecessor Institutions, EUA IN23/4/1/1/1. On the transition of the AFRC, ABRO, the IAPGR and the Roslin Institute from a mainly agricultural to an increasingly biomedical focus – embodied in the change of name of the research council to BBSRC in 1994 – see Myelnikov, 2017 and García-Sancho, 2015.
